# PtrA regulates prodigiosin synthesis and biological functions in *Serratia marcescens* FZSF02

**DOI:** 10.3389/fmicb.2023.1240102

**Published:** 2023-09-15

**Authors:** Junjie Lin, Yanshuang Yu, Ke Zhao, Jie Zhao, Christopher Rensing, Jichen Chen, Xianbo Jia

**Affiliations:** ^1^Institute of Soil and Fertilizer, Academy of Agricultural Sciences/Fujian Key Laboratory of Plant Nutrition and Fertilizer, Fuzhou, China; ^2^College of Life Sciences, Fujian Agriculture and Forestry University, Fuzhou, China; ^3^College of Resources and Environment, Institute of Environmental Microbiology, Fujian Agriculture and Forestry University, Fuzhou, Fujian, China

**Keywords:** PtrA, stress tolerance, prodigiosin synthesis, cell motility, *Serratia marcescens*

## Abstract

*Serratia marcescens* is a gram-negative bacterium that is able to produce many secondary metabolites, such as the prominent red pigment prodigiosin (PG). In this work, a *ptrA*-disrupted mutant strain with reduced PG production was selected from Tn*5* transposon mutants. RT–qPCR results indicated that *ptrA* promoted elevated transcription of the *pig* gene cluster in *S. marcescens* FZSF02. Furthermore, we found that *ptrA* also controls several other important biological functions of *S. marcescens*, including swimming and swarming motilities, biofilm formation, hemolytic activity, and stress tolerance. In conclusion, this study demonstrates that *ptrA* is a PG synthesis-promoting factor in *S. marcescens* and provides a brief understanding of the regulatory mechanism of *ptrA* in *S. marcescens* cell motility and hemolytic activity.

## 1. Introduction

*S. marcescens* is a widely distributed gram-negative, rod-shaped bacterium found in soil, water, plant surfaces, and insects (Grimont and Grimont, 1978) and is used for the production of many high-value products, including prodigiosin (Han et al., 2021), serratamolide (Clements et al., 2019), acetoin (Bai et al., 2015), althiomycin (Gerc et al., 2012), and 2,3-butanediol (Zhang et al., 2010). Therefore, *S. marcescens* has become an important microbial strain for industrial applications. In addition, *S. marcescens* is also a well-known opportunistic pathogen that infects plants, invertebrates, and vertebrates (Mahlen, 2011). *S. marcescens* has been shown to cause septicaemia (Wilfert et al., 1968), empyema (Nakashima et al., 1987), meningitis (Psautter et al., 1984) and other diseases.

Prodigiosin is a tripyrrole red secondary metabolite of microbial origin that has antibacterial (Lapenda et al., 2015), algicidal (Wei et al., 2020), antimalarial (Papireddy et al., 2011), immunomodulatory (Chang et al., 2011), and antitumor activities (Zhang et al., 2005; Pan et al., 2012). The synthesis of prodigiosin by *S. marcescens* is mainly controlled by 14 genes, including *pigA-N*, located in the same operon (Williamson et al., 2006). Environmental factors, such as nutrient deprivation, temperature, oxygen, pH, light, ionic strength, and phosphate availability, were also shown to affect prodigiosin generation (Solé et al., 1994; Bennett and Bentley, 2000; Slater et al., 2003; Giri et al., 2004; Williamson et al., 2006; Ryazantseva et al., 2012). Although the synthetic process of prodigiosin in *S. marcescens* has been elucidated in recent years, the understanding of the regulatory mechanisms responsible for prodigiosin biosynthesis in *S. marcescens* is still limited.

The protein encoded by *ptrA* is the metalloendopeptidase pitrilysin (Anastasi and Barrett, 1995). Pitrilysin has been generated by heterologous expression in *Escherichia coli* and was able to degrade small molecular polypeptides, such as insulin B chain, but could not degrade proteins, so it was also called an “oligopeptidase” type of endopeptidase (Anastasi et al., 1993).

In this study, the prodigiosin-producing wild-type strain *S. marcescens* FZSF02 was isolated from soil samples (Lin et al., 2019). In the work, a *ptrA*-disrupted mutant strain with reduced PG production was selected from Tn*5* transposon mutants and Δ*ptrA* was constructed in the wild-type strain FZSF02 with the homologous recombination method. The mechanism for positive regulation of prodigiosin production by PtrA protein was explored. Interestingly, PtrA also regulated swarming and swimming motilities, biofilm formation, hemolytic activity and stress resistance in *S. marcescens*. Our study data showed a novel regulator PtrA, which is important for various cellular processes in *S. marcescens*.

## 2. Materials and methods

### 2.1. Bacterial strains, plasmids, and culture conditions

*S. marcescens* FZSF02 is a prodigiosin-producing strain isolated from a soil sample (Lin et al., 2019). Mutant E35 displays reduced production of prodigiosin and was isolated from a Tn*5* transposon insertion mutant library of strain FZSF02 (Jia et al., 2021). *S. marcescens* strains were incubated in Luria–Bertani (LB) medium (10 g tryptone, 5 g yeast extract and 5 g NaCl per liter) at 27°C or 37°C.

Antibiotics were added when required at the following final concentration: kanamycin (100 μg/mL). The bacterial strains and plasmids used in this study are listed in Table 1. The primers used in this study are listed in Table 2.

**Table 1:** Bacterial strains used in this study.

Strain	Description	Source or reference
*S. marcescens* FZSF02	*S. marcescens* wild-type strain	This study
E35	Wild-type strain FZSF02 with in-frame deletion of *ptrA* gene, Km^R^	Jia et al. (2021)
Δ*ptrA*	*ptrA* deletion mutant of *S. marcescens* FZSF02, Km^R^	This study
Δ*swrW*	*swrW* deletion mutant of *S. marcescens* FZSF02, Km^R^	This study

**Table 2:** Primers used in this study.

Primers	Sequences (5′ → 3′)	Function
KanproF1	TCTCAACCATCATCGATGAATTGT	Amplification of the Kan^R^ gene
KanR	TTAGAAAAACTCATCGAGCATCA	
PtrA KnocF	ATGCGCAGACAGTTGGCCCG	Forward primer for amplifying the upstream homologous arm of *ptrA*
PtrA overR	TTCATCGATGATGGTTGAGAGACGGAAGATCAGCTGGCGC	
PtrA overF	TGCTCGATGAGTTTTTCTAACCAACCCTTGCTTCTGCAGC	Reverse primer for amplifying the upstream homologous arm of *ptrA*
PtrA KnocR	TGGCGTCGCCACTTTGCGCG	
PtrA midF	ATCACGCGCGACATGGACTAC	Identification of Δ*ptrA* deletion mutant
PtrA midR	GATTGCGCGCGGAATCCATGG	
16SF	CGTTACTCGCAGAAGAAGCA	reference gene of RT–qPCR
16SR	TCACCGCTACACCTGGAA	
PigAF	CGCCATCTTCCACGATTCAA	RT–qPCR of *pigA* gene
PigAR	CATTAGCCGACACTGTTCCA	
PigFF	CACGGTATTCGGCGATGAC	RT–qPCR of *pigF* gene
PigFR	CACGGTGTTGCGAGAAGT	
PigNF	CGGTTACCCTGGTCTATTG	RT–qPCR of *pigN* gene
PigNR	TGTCAGCACGATGTTCAT	
FlgHF	CGCCGATATGGACATTTC	RT–qPCR of flgH gene
FlgHR	GTAATGGTGCCGTTGAAG	
SwrWF	GTGTCCGCTTATTCCCTGACG	RT–qPCR of *swrW* gene
SwrWR	TCAAGGAAGGTTGCCTAGCATC	

### 2.2. Construction of the *ptrA* gene deletion mutant

*ptrA* was screened and identified from previously constructed Tn*5* transposon insertion mutant library of strain FZSF02 (Jia et al., 2021). The homologous recombination method was used to inactivate genes. Briefly, the forward homologous sequence of approximately 1,000 bp was amplified with primers PtrA knocF and PtrA overR, and the backward homologous sequence of approximately 1,000 bp was amplified with primers PtrA overF and PtrA knocR. The kanamycin resistance gene was amplified with primers KanproF and KanR; these three sequences were spliced by overlapping PCR. The overlapping PCR products were purified and then transformed into *S. marcescens* FZSF02 by using Gene Pulser Xcell (Bio-Rad) for electroporation for subsequent homologous recombination deletion of *ptrA*. Then, colonies containing deletions of the *ptrA* gene were selected on LB agar medium containing kanamycin at 27°C and identified by PCR, and Δ*ptrA* was finally confirmed by sequence comparison.

### 2.3. Prodigiosin production assays

Determination of the prodigiosin yield of strains FZSF02 (WT) and Δ*ptrA* was carried out with a modified method as reported previously (Kalivoda et al., 2010). Briefly, single colonies of WT and Δ*ptrA* were selected from plates and inoculated into LB liquid medium overnight culture at 180 r/min at 37°C. One milliliter of the broth was transferred into 50 mL of fresh LB liquid medium and cultured with shaking at 27°C and 180 r/min for 24 h. The broth was properly diluted with acidified methanol (4 mL of 1 mol/L HCl and 96 mL of methanol) and shaken vigorously; after 10 min of standing, the mixture was centrifuged at 8,000 × g for 10 min, and the supernatant was removed for prodigiosin quantification. Prodigiosin production can be evaluated by the absorbance value at 535 nm (de Araújo et al., 2010). Optical densities of cultures were measured at 535 and 600 nm wavelength at time intervals of 0, 3, 6, 9, 12, 15, 18, 21, 24, 36 and 48 h, relative prodigiosin production of different strains was calculated by the value of A_535_/OD_600_, where the OD_600_ values of the fermentation broth represent the biomass of the strains. Experiments were independently replicated three times.

### 2.4. Growth curve measurements

To analyze the growth curve of strains FZSF02 and Δ*ptrA*, the exponential-phase cells (OD_600_ of 1.0) of these two strains were inoculated in fresh LB medium at a 2% inoculation volume. Optical densities of cultures were measured at 600 nm wavelength at time intervals of 0, 3, 6, 9, 12, 15, 18, 21, 24, 36, and 48 h, and the growth curves were plotted as values at OD_600_ versus the incubation time. Experiments were independently replicated three times.

### 2.5. RNA extraction and quantitative real-time PCR

The samples were treated with the TransZol Up Plus RNA Kit (ER501; TransGen, Beijing, China) for total RNA extraction, and the total bacterial RNA was subjected to reverse transcription using FastKing gDNA Dispelling RT SuperMix (KR118; Tiangen, Beijing, China).

The RT-qPCRs were carried out in a final volume of 20 μL according to the manufacturer’s instructions, using 1 μL of the cDNA dilution as template, and were mixed with 0.4 μL of forward and reverse primers. The mixture was then exposed to RT–qPCR analysis using *TransStart*^®^ Green qPCR SuperMix (AQ101-02; TransGen, Beijing, China) in the reaction in QuantStudio™ 6 Flex Real-Time PCR System Software (Applied Biosystems). Gene expression levels were measured using the 16S rRNA gene as a control using the 16SF and 16SR primers (Table 2). Gene expression levels of mutant strains were determined using the 2^−ΔΔCT^ method with the relative fold-difference expression against the wild-type strain. The RT–qPCR from three biological replicates was analyzed, and three technical replicates were performed.

### 2.6. Motility assays

Swimming and swarming motility assays were performed by dropping 2 μL of the exponential phase cells (OD_600_ of 1.0) of both the FZSF02 and the Δ*ptrA* strain onto the center of LB media plates with 0.3% (Swimming) or 0.7% (Swarming) BD agar (BD: Becton, Dickinson and Company), respectively (Soo et al., 2008, 2014). The swarming and swimming zones were observed, and the migration diameter was measured after incubation at 27°C for 12 h. Each group was independently repeated three times.

### 2.7. Biofilm analysis

The ability of the strains to form biofilms was assayed according to a previously reported method (Shanks et al., 2007). In detail, 1 mL of exponential-phase broth (OD_600_ of 1.0) was added to 50 mL of liquid LB medium, and then 200 μL of the mixed broth was added into the wells of 96-well microtiter plates (No. 655180. Greiner Bio-One). After 48 h of nonshaking incubation at 27°C, the optical density at OD_600_ was measured and recorded. After that, the biofilm was gently washed with distilled water five times and stained with 200 μL of crystal violet solution (0.1% w/v) for 15 min. Thereafter, the biofilm was gently washed with distilled water five times, and crystal violet was extracted with 200 μL of anhydrous ethanol. Finally, the optical density of the biofilm was measured at a wavelength of 595 nm using the SPECTRA MAX190 microplate reader (Molecular Devices). Biofilm yield ability was calculated by comparing the ratio of the value at OD_595_ to the value at OD_600_.

### 2.8. Stress tolerance assays

Fresh broth of FZSF02 and Δ*ptrA* strains was adjusted to a density of OD_600_ 1.0 with sterile water and used for the following tests. For H_2_O_2_, acid and osmotic tolerance assays, the broth was serially diluted (10-fold) with sterile water, and then 2 μL of the solutions were spotted onto LB agar medium containing 2 mM H_2_O_2_, 175 mM acetic acid and 3 M NaCl. For heat tolerance assays, the bacteria were treated at 50°C for 20 min, serially diluted (10-fold) with sterile water, and finally spotted onto LB agar medium. All plates were incubated for 24 h at 27°C.

Meanwhile, the CFU counting method was used to quantitatively evaluate the tolerance ability for H_2_O_2_, acid, osmotic and heat. For H_2_O_2_, the bacterial broth was treated with 2 mM H_2_O_2_ (prepared with 0.9% NaCl) for 5, 15, 30, and 45 min. For heat tolerance assays, the bacterial broth was treated at 50°C for 0, 5, 10, 15, and 20 min. For acid tolerance assays, the bacterial broth was treated with 175 mM acetic acid (prepared with 0.9% NaCl) for 0, 5, 10, 15 and 20 min. For osmotic tolerance assays, the bacterial broth was treated with 3 M NaCl for 0, 5, 10, 15 and 20 min. All treatments above were diluted (10-fold), spread on LB agar plates and incubated for 24 h at 27°C followed by CFU counting.

### 2.9. Catalase activity

Catalase activity was performed as previously described (de Ondarza, 2017). Briefly, the strains were cultured in liquid LB medium at 27°C and 180 r/min for 24 h. The cells in the broth were crushed by ultrasound and centrifuged at 4,000 r/min at 4°C for 10 min, and the subsequently obtained supernatant was a crude enzyme solution of catalase. To assay the catalase activity, 2.9 mL of 2 mM H_2_O_2_ (prepared by adding 0.15 mL of 30% H_2_O_2_ to 50 mL of PBS buffer) and 0.1 mL of diluted enzyme solution were mixed, and recording was immediately carried out at 240 nm every 20 s for a total time of 2 min. Catalase activity was calculated by the following formula: (ΔA240/min * dilution ratio)/(43.6/mol/min * sample volume * 0.001).

### 2.10. Hemolytic activity assay

Hemolytic activity was assayed as previously described (Di Venanzio et al., 2014; Ridder et al., 2021). Briefly, 2 μL of exponential-phase culture (OD_600_ = 1.0) was spotted on a blood agar plate (Hunan BKMAM Biotechnology Co., Ltd) and cultured at 27°C and 37°C for 5 days, and the diameters of the hemolytic transparent zones were measured. Experiments were independently repeated three times.

### 2.11. Statistical analysis

Student’s *t* tests or one-way ANOVA were used to compare significant differences between experimental groups, while multiple comparisons were conducted using one-way ANOVA with GraphPad Prism software. All experiments were independently replicated at least three times in this study.

## 3. Results and discussion

### 3.1. Identification of a prodigiosin synthesis activator PtrA

**Figure 1:**
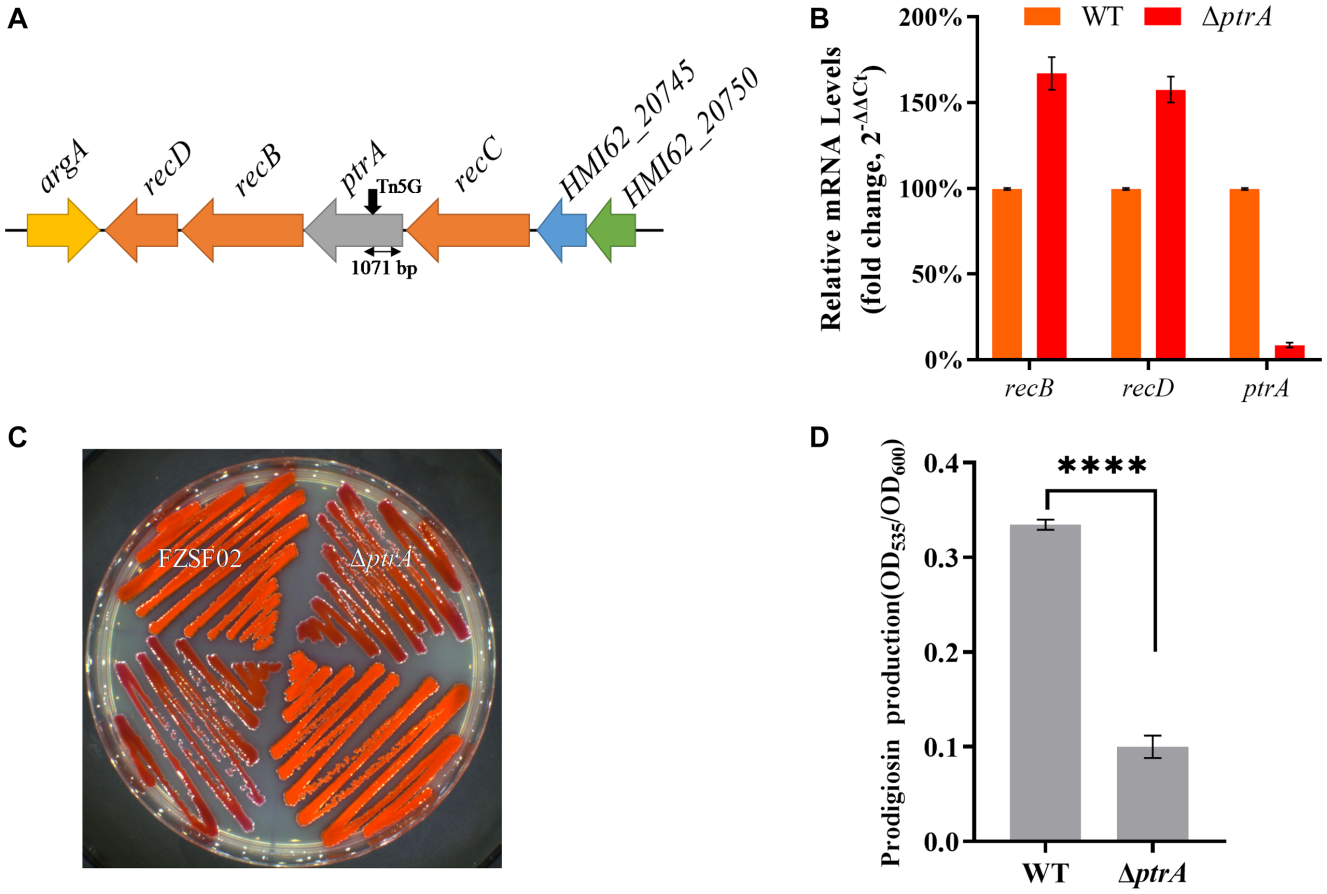
PtrA positively regulates the synthesis of prodigiosin in strain FZSF02. **(A)** The genetic locus identified in mutant E35. The panel shows the genetic map of the disrupted gene *ptrA* and its surroundings, and the black arrow indicates the position of the Tn*5* insertion. **(B)** RT–qPCR analysis of the relative expression levels of *recB* and *recD* in WT and mutant Δ*ptrA*. **(C)** Phenotype of the *ptrA* inactivated mutant strain Δ*ptrA*. **(D)** Analysis of prodigiosin production in FZSF02 and Δ*ptrA*. FZSF02 is wild-type *S. marcescens*, and Δ*ptrA* is the *ptrA*-disrupted mutant. For **(C)**, the experiments were independently replicated three times. Error bars represent standard deviations. Pairwise comparisons were performed using Student’s *t* test, while multiple comparisons were conducted using one-way ANOVA, ****p < 0.0001.

A mutant E35 with positively regulated synthesis of prodigiosin was isolated from a Tn*5* transposon mutant library constructed with *Serratia marcescens* FZSF02. Transposon Tn*5* was inserted in the coding region between 1,071 bp and 1,072 bp of the *HMI62_20735* gene in E35, which encodes a protein with 100% identity to predicted PtrA (GenBank accession number QJU42755.1) (Figure 1A). PtrA of FZSF02 displayed 66.84, 66.84 and 66.53% similarities with *E. coli* CFT073 (Q8CVS2.1), *Shigella flexneri* (Q83QC3-1) and *Salmonella enterica* sp. LT2 (Q8ZMB5.1), respectively (Supplementary Figure S1).

*ptrA* appeared to be the first gene in an operon, so polar effects are a concern. The effect of polar transcription on *recB/D* was verified by RT–qPCR, and the results showed that the relative expression levels of *recB* and *recD* in Δ*ptrA* were upregulated by 1.67-fold and 1.58-fold, respectively, compared to the WT strain (Figure 1B). There was no significant difference, suggesting that the PtrA mutation was responsible for the phenotypes.

To further confirm the function of PtrA, a *ptrA* in-frame deletion strain Δ*ptrA* was constructed (Figure 1C). The prodigiosin production of the mutant *ptrA* was only 0.3 times that of the wild-type strain FZSF02 (*p* < 0.0001, Figure 1D) when incubated for 48 h. The results suggested that *ptrA* encoded a protein, PtrA, that promoted prodigiosin synthesis in strain FZSF02.

### 3.2. PtrA affects the transcription level of the prodigiosin-associated *pigA*-*pigN* gene cluster

**Figure 2:**
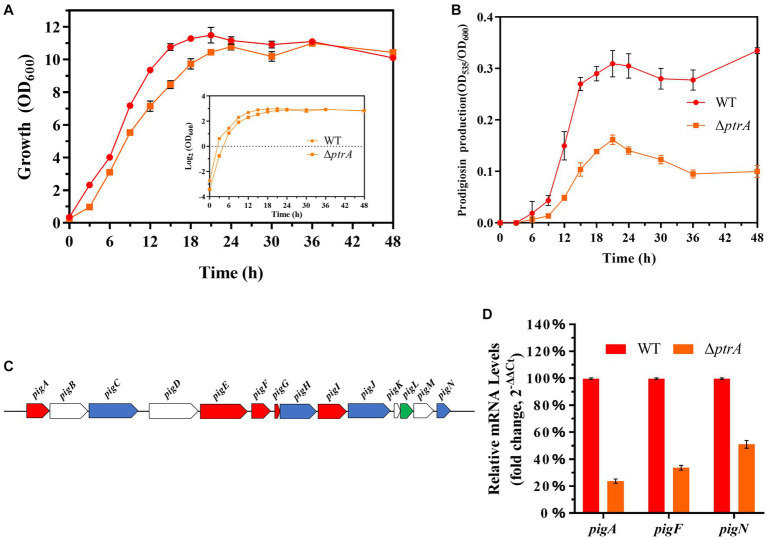
PtrA promotes the transcription of the *pig* gene cluster. **(A)** Growth curves of WT and Δ*ptrA* strains by measuring OD_600_. **(B)** Unit cell production of prodigiosin of WT and Δ*ptrA* strains at all time points. **(C)** The panel shows the genetic map of the *pig* gene cluster. **(D)** RT–qPCR analysis of the relative expression levels of the *pigA, pigF, and pigN* genes in the *pig* gene cluster in WT and mutant Δ*ptrA*. For **(A,B,D)**, the experiments were independently replicated three times. Error bars represent standard deviations.

To study the effect of PtrA on bacterial growth ability, growth curves of WT and mutant Δ*ptrA* were determined (Figure 2A). Compared to the wild-type strain FZSF02, the growth of Δ*ptrA* was significantly slower from 0 to 36 h, which may be due to the synthesis of primary metabolites being decreased in Δ*ptrA*. After 36 h, these two strains reached almost the same final biomass. At each sampling point, the OD_535_/OD_600_ value of Δ*ptrA* was much lower than that of the wild-type strain (*p* < 0.0001, Figure 2B), which means that the ability of Δ*ptrA* to synthesize prodigiosin was significantly reduced, and this decrease may be caused by the decreased growth ability and the low expression level of *pig* genes at 0–36 h. While after 36 h, this decrease was not due to a decrease in growth ability but probably due to lower expression levels of *pig* genes.

The synthesis of prodigiosin by *S. marcescens* is mainly controlled by 14 genes, including *pigA* through *pigN*, which are located on the same operon (Figure 2C; Williamson et al., 2006). The results of real-time quantitative PCR (RT–qPCR) showed that the relative expression levels of *pigA*, *pigF*, and *pigN* in Δ*ptrA* were reduced by 4.20-, 2.73-, and 1.96-fold, respectively (Figure 2D). These results suggest that PtrA promoted the synthesis of prodigiosin by positively regulating the expression of *pig* genes.

### 3.3. PtrA positively controls cell motility

**Figure 3:**
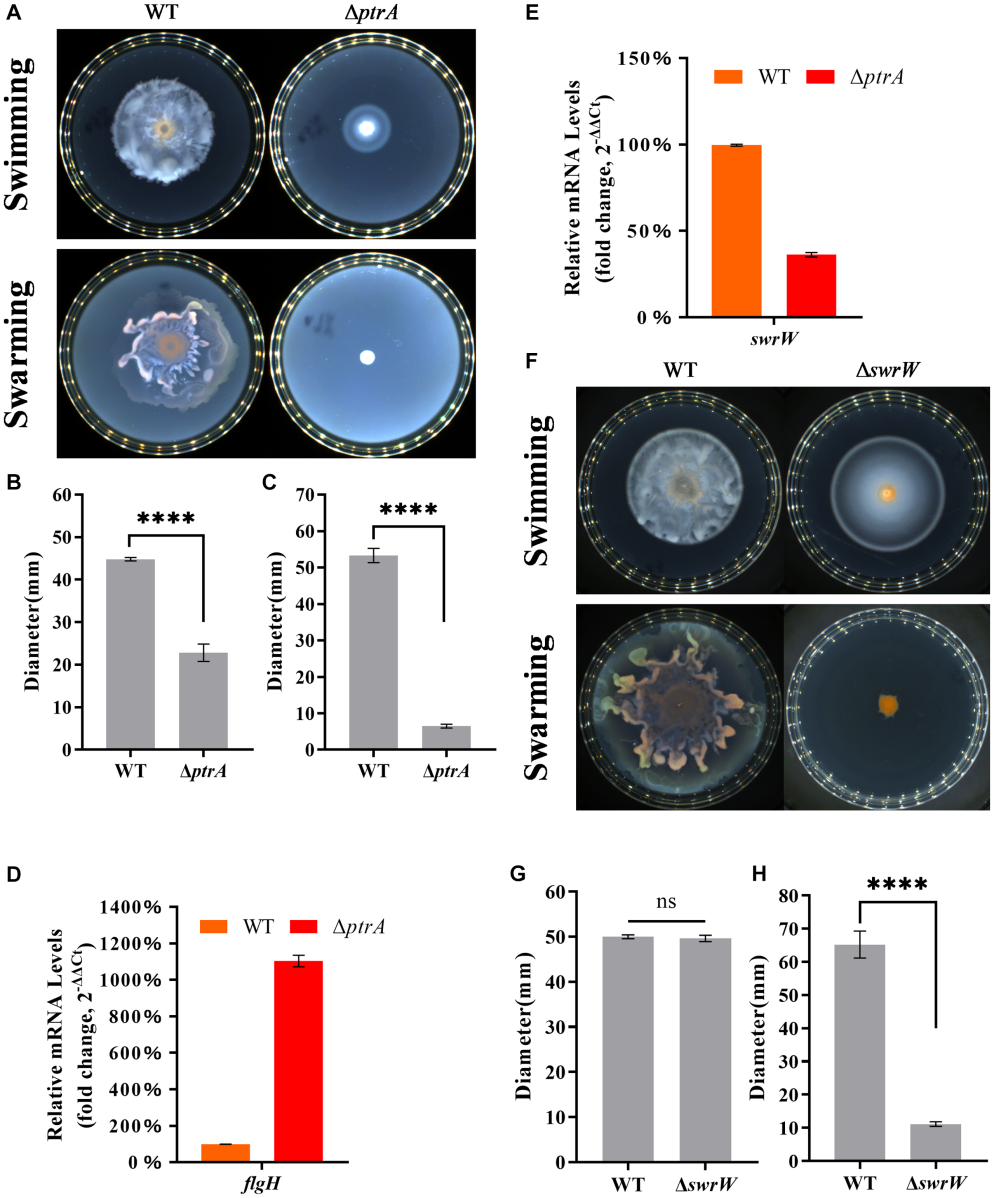
PtrA regulates cell motility in *S. marcescens*. **(A)** Swimming and swarming assay for WT and Δ*ptrA*. **(B)** Colony diameter determination of WT and Δ*ptrA* for the swimming assay. **(C)** Colony diameter determination of WT and Δ*ptrA* for the swarming assay. **(D)** RT–qPCR analysis of the relative expression levels of *flgH* in flagellar synthesis in WT and mutant Δ*ptrA*. **(E)** RT–qPCR analysis of the relative expression levels of *swrW* in WT and mutant Δ*ptrA*. **(F)** Swimming and swarming assay for WT and Δ*swrW*. **(G)** Colony diameter determination of WT and Δ*swrW* for the swimming assay. **(H)** Colony diameter determination of WT and Δ*swrW* for the swarming assay. For **(B–E,G,H)**, the experiments were independently replicated three times. Error bars represent standard deviations. Pairwise comparisons were performed using Student’s *t* test, while multiple comparisons were conducted using one-way ANOVA, ****p < 0.0001.

Previous studies have shown that swimming and swarming are two modes of motility found in *S. marcescens* (Alberti and Harshey, 1990). In this study, the swimming distance of the mutant Δ*ptrA* was significantly reduced compared to that of the wild-type strain FZSF02 when incubated on plates containing 0.3% (m/V) agar (Figure 3A, top; Figure 3B). For the swarming test, wild-type FZSF02 displayed an extended migration distance, but Δ*ptrA* showed no migration distance (Figure 3A, bottom; Figure 3C), and the results indicated that PtrA positively affected the swimming and swarming functions of FZSF02.

The motility of bacteria can be divided into swimming and swarming, and the flagellum has been shown to contribute to the motility of *S. marcescens* (Pan et al., 2020). To further analyze the relationship between PtrA and flagellum, the key gene *flgH* in the process of flagellar synthesis was selected for further analysis. Surprisingly, the RT–qPCR results showed that the relative expression level of *flgH* in Δ*ptrA* was upregulated by 11.03-fold compared to the WT strain (Figure 3D). Surprisingly, Δ*ptrA* displayed decreased motility but significantly upregulated *flgH* expression levels. However, it has been reported that serrawettin W1 (serratamolide) was distinguished from the flagellum in contributing to the swarming motility, while PigP mediated swarming motility of *S. marcescens* through control of serratamolide biosynthesis (Shanks et al., 2013). Serrawettin W1 was reported to act as a biosurfactant, reducing surface tension when *S. marcescens* swarms on a surface, and the gene *swrW* is required for biosynthesis of the biosurfactant serratamolide (Matsuyama et al., 1992; Li et al., 2005). To investigate whether *swrW* is downregulated in Δ*ptrA*, the expression levels of *swrW* (HMI62_23130 gene) (GenBank accession number QJU42029.1) were assayed by RT–qPCR. The results showed that the relative expression levels of *swrW* in Δ*ptrA* were downregulated by 2.76-fold (Figure 3E). At the same time, we constructed a *swrW* (HMI62_23130 gene) deletion mutant Δ*swrW* and performed swimming and swarming experiments. The results showed that the swimming movement migration distance of the mutant Δ*swrW* was no significant difference (Figure 3F, top; Figure 3G) and the swarming movement migration distance of the mutant Δ*swrW* was significantly reduced (Figure 3F, bottom; Figure 3H). These results demonstrated that *ptrA* positively regulated the swarming motility of *S. marcescens* FZSF02 by regulating the expression level of *swrW* but not *flgH*. The reason for *flgH* showing significantly upregulated expression levels in Δ*ptrA* remains to be elucidated.

### 3.4. PtrA regulates biofilm formation and serrawettin W1 biosynthesis

**Figure 4:**
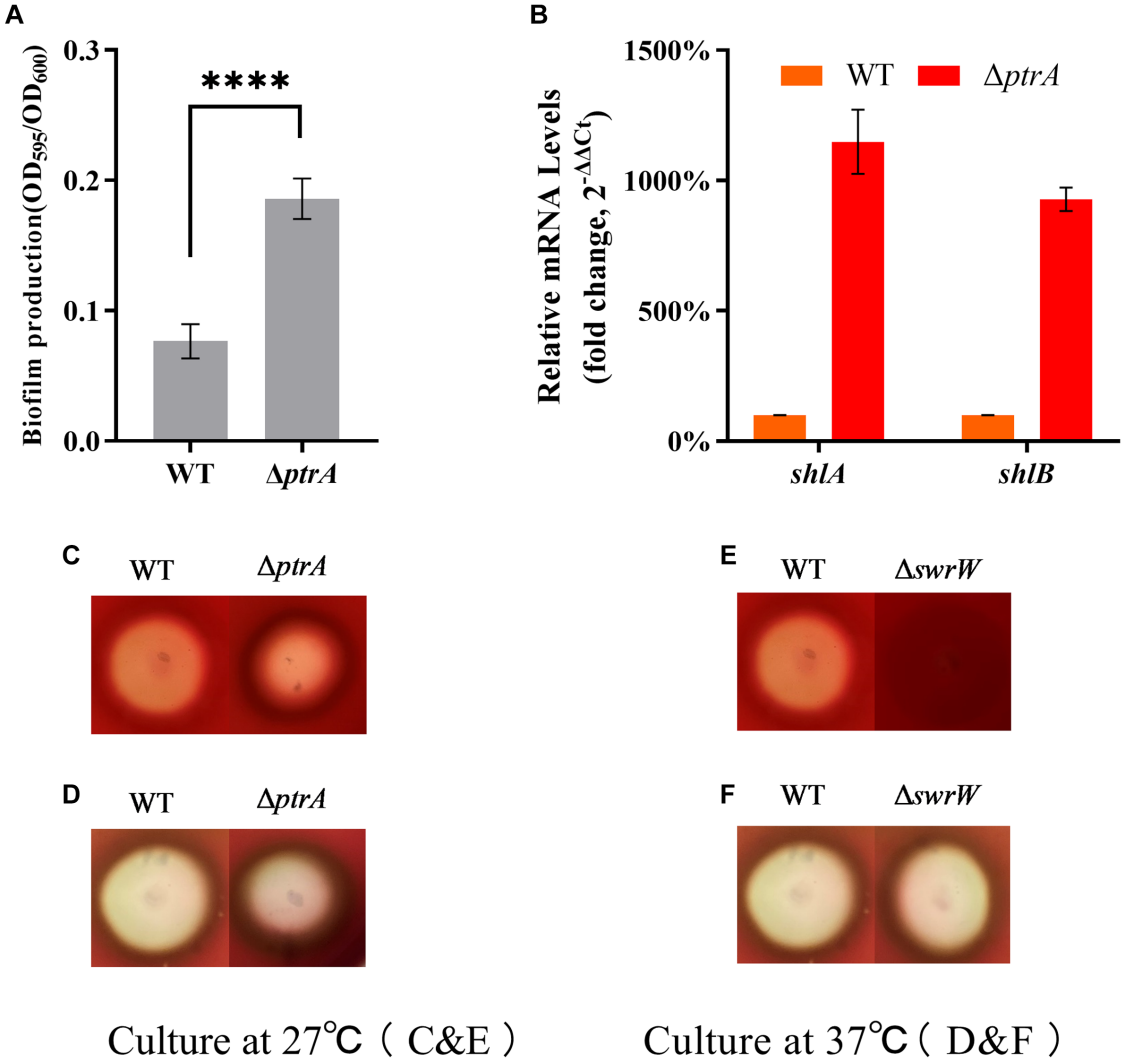
PtrA does not contribute to biofilm formation but contributes to serrawettin W1 biosynthesis in *S. marcescens*. **(A)** PtrA negatively regulates biofilm formation in WT. **(B)** RT–qPCR analysis of the relative expression levels of *shlA* and *shlB* in WT and mutant Δ*ptrA*. **(C)** Hemolytic transparent circles of WT and Δ*ptrA* at 27°C. **(D)** Hemolytic transparent circles of WT and Δ*ptrA* at 37°C. **(E)** Hemolytic transparent circles of WT and Δ*swrW* at 27°C. **(F)** Hemolytic transparent circles of WT and Δ*swrW* at 37°C. For **(A)**, the experiments were independently replicated three times. Error bars represent standard deviations. Pairwise comparisons were performed using Student’s *t* test, while multiple comparisons were conducted using one-way ANOVA, ****p < 0.0001.

*S. marcescens* is an opportunistic bacterium, and biofilm formation is a major requirement of pathogenesis. Biofilm formation in *S. marcescens* was linked to its ability to colonize, persist, and proliferate on either biological or inert surfaces (Choe et al., 2012). Therefore, we investigated whether PtrA was able to affect the ability to form biofilms in *S. marcescens* FZSF02. The results showed that biofilm production of mutant Δ*ptrA* was significantly increased compared to FZSF02 (*p* < 0.0001, Figure 4A), indicating that PtrA negatively regulates *S. marcescens* biofilm synthesis.

Previous research has shown hemolytic activity in *S. marcescens* (Braun et al., 1985). Two genes, *shlA* and *shlB*, encode a hemolysin and an accessory protein necessary for activity, respectively, and both gene products are present in the outer membrane (Poole et al., 1988). The above results show that the biofilm production of Δ*ptrA* is higher than that of WT, which means that the hemolysis of Δ*ptrA* may be higher than that of WT. To test this idea, we performed hemolysis experiments on blood agar plates. The results showed that the hemolytic transparent zones of Δ*ptrA* were significantly reduced compared to the WT at 27°C, indicating that the hemolytic activity was significantly reduced (Figure 4B); however, there was no significant difference in the hemolytic transparent zones between Δ*ptrA* and WT at 37°C (Figure 4C).

To verify whether the hemolytic changes were caused by the changes in the expression levels of *shlA* and *shlB*, RT–qPCR analysis was performed on *shlA* and *shlB*. Surprisingly, the results showed that the relative expression levels of *shlA* and *shlB* in Δ*ptrA* were upregulated by 11.49-fold and 9.28-fold, respectively, compared to the WT strain (Figure 4D). This indicates that the hemolysis of *S. marcescens* FZSF02 is not regulated by *shlA* and *shlB*. A literature review found that serrawettin W1 produced by *S. marcescens* is a surface-active exolipid with various functions supporting the behaviors of bacteria in surface environments (Matsuyama et al., 1989). Serrawettin W1 is a hemolytic factor produced by *S. marcescens* (Shanks et al., 2012), and PigP mediated hemolysis through control of serratamolide biosynthesis (Shanks et al., 2013). The hemolysis of WT and Δ*swrW* was determined, and the results showed that the hemolytic activity of Δ*swrW* also significantly decreased at 27°C (Figure 4E) and showed no significant change compared to that of the WT at 37°C (Figure 4F). A previous study showed that, similar to prodigiosin, the production of serrawettin W1 by *S. marcescens* is also temperature-regulated. That is, a positive product was produced at 27°C and inhibited at 37°C (Matsuyama et al., 1986). Overall, PtrA promoted serrawettin W1 biosynthesis and promoted hemolysis activity at 27°C.

### 3.5. PtrA plays an important role in stress tolerance in *S. marcescens*

**Figure 5:**
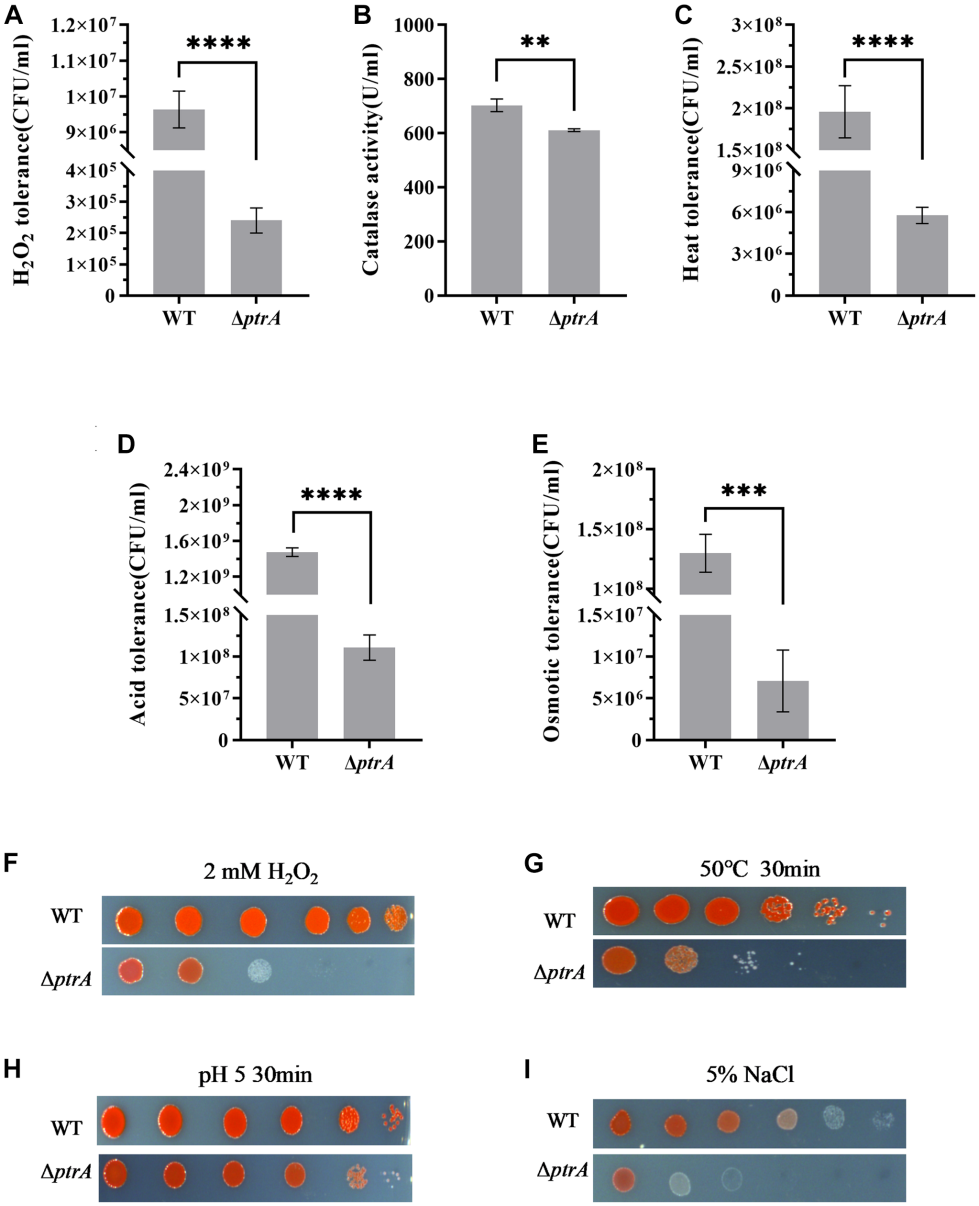
Influence of PtrA on H_2_O_2_ tolerance, heat tolerance, oxidative tolerance and acid tolerance. **(A)** H_2_O_2_ tolerance CFU counting of WT and Δ*ptrA*. **(B)** PtrA positively regulates catalase activity in WT. **(C)** Heat tolerance CFU counting of WT and Δ*ptrA*. **(D)** Acid tolerance CFU counting of WT and Δ*ptrA*. **(E)** Osmotic tolerance CFU counting of WT and Δ*ptrA*. **(F)** H_2_O_2_ tolerance spotting assay of WT and Δ*ptrA*. **(G)** Heat tolerance spotting assay of WT and Δ*ptrA*. **(H)** Acid tolerance spotting assay of WT and Δ*ptrA*. **(I)** Osmotic tolerance spotting assay of WT and Δ*ptrA*. For **(A–D)**, the experiments were independently replicated three times. Error bars represent standard deviations. Pairwise comparisons were performed using Student’s *t* test, while multiple comparisons were conducted using one-way ANOVA, ****p < 0.0001.

Environmental stresses are usually active during the process of microbial fermentation and have a significant influence on microbial physiology (Guan et al., 2017). Hydrogen peroxide generated during oxidative stress is known to damage proteins, nucleic acids and cell membranes and has been implicated in cancer, aging, and several chronic neurodegenerative diseases and therefore presents a major challenge for aerobic organisms (Daroui et al., 2004). In this study, the H_2_O_2_ tolerance of Δ*ptrA* was significantly decreased and only 2.49% of that of the WT survived (*p* < 0.0001, Figures 5A,F), indicating that PtrA was involved in cellular tolerance to H_2_O_2_. In bacteria, H_2_O_2_ is eliminated by catalase and peroxidase. To confirm whether the significantly reduced H_2_O_2_ tolerance of the mutant Δ*ptrA* was due to the reduced production of catalase, catalase activities of WT and Δ*ptrA* were assayed. The results showed that compared with WT, the catalase activity of Δ*ptrA* was significantly decreased (*p* < 0.01, Figure 5B), indicating that one of the reasons for the significantly decreased H_2_O_2_ tolerance of Δ*ptrA* was the decreased production of catalase.

Adaptation to changes in ambient temperature is a common feature of all living organisms (Richter et al., 2010). The heat tolerance of Δ*ptrA* was significantly decreased and only 2.49% of that of the WT in survival (*p* < 0.0001, Figures 5C,G), indicating that PtrA is involved in the heat tolerance of *S. marcescens*.

To meet sudden and potentially lethal challenges, microorganisms must have effective mechanisms to withstand a variety of environmental stressors, acid resistance being one of the most common features (Waterman and Small, 2003). Therefore, the acid tolerance of Δ*ptrA* was investigated, and the results showed that the acid tolerance of Δ*ptrA* was significantly decreased and only 7.52% of that of the WT in survival (*p* < 0.0001, Figures 5D,H), strongly suggesting that PtrA plays an important role in the acid resistance of *S. marcescens*.

Bacteria inhabit natural and artificial environments with diverse and fluctuating osmolalities, one of the most ubiquitous stress factors encountered by microorganisms in their habitats (Wood, 2011). To investigate the effect of PtrA on the osmotic stress tolerance of *S. marcescens*, the osmotic tolerance of the FZSF02 and Δ*ptrA* mutant strains was analyzed. The results showed that the osmotic tolerance of Δ*ptrA* was significantly reduced, and only 5.48% of that was conferred by the WT (*p* < 0.001, Figures 5E,I), indicating that PtrA positively influences the osmotic tolerance of *S. marcescens*.

Although synthetic biology has experienced significant development, the efficiency of modifying and improving complex traits, such as stress tolerance and growth rates, is difficult to achieve because of the complex metabolic and regulatory networks involved. Therefore, adaptation is still a widely used method to improve microbial performance (Sandberg et al., 2019). The stress tole-rance in response to hydrogen peroxide, high temperature, acidic environment and high osmotic fluctuations was significantly reduced in Δ*ptrA* mutants, displaying a significant reduction in the environmental adaptability of Δ*ptrA*. The adaptability of microorganisms compensates for the lack of knowledge regarding the microbial ability to utilize nonpreferred substrates by rapidly improving the ability of microorganisms to utilize nonpreferred substrates (Tan et al., 2022). Therefore, a decrease in the adaptability of Δ*ptrA* means a decrease in its viability. In other words, *ptrA* might be a potential gene for synthetic biology applications to improve the performance of various vital characteristics in bacterial strains.

The homolog of PtrA is Protease III in *E. coli* K-12, and it is devoid of activity toward aminopeptidase, carboxypeptidase, or esterase substrates but rapidly degrades small proteins (Cheng and Zipser, 1979). Studies on the biological functions of cell motility, hemolysis and stress adaptation have not been reported, to the best of our knowledge. Therefore, it could not be compared with other bacteria to determine whether PtrA in other bacteria also controlled the same cellular processes in *Serratia marcescens*.

## 4. Conclusion

In this paper, we studied the effect of PtrA on prodigiosin synthesis and other biological functions of *S. marcescens* FZSF02. Growth ability and prodigiosin significantly decreased in Δ*ptrA*, and the reason for the decrease in prodigiosin production was not related to biomass; rather, it was associated with decreased expression of the *pig* gene cluster in Δ*ptrA*. Mutation of ptrA negatively influenced the mobility and hemolytic activity of FZSF02 by lowering the expression level of *swrW*. The results also showed that a deletion of PtrA is not conducive to the survival of *S. marcescens* in environments with elevated levels of H_2_O_2_, heat, acid and osmotic stress. This might be the first time the function of PtrA was studied in detail. This study provided new insights into PtrA-dependent regulation of prodigiosin biosynthesis. Multiple effects on the growth, mobility, hemolytic activity, and stress adaptation of FZSF02 indicated that PtrA might be key for improving the performance of industrial microbial strains in the future.

## Data availability statement

The original contributions presented in the study are included in the article/Supplementary material, further inquiries can be directed to the corresponding author.

## Author contributions

JL: investigation, formal analysis, methodology, and writing – original draft. YY: conceptualization, methodology, investigation, and writing – original draft. KZ: data curation, conceptualization, and validation. JZ: formal analysis and investigation. CR: investigation. JC: formal analysis and funding acquisition. XJ: conceptualization, methodology, writing – review and editing, funding acquisition, and project administration. All authors contributed to the article and approved the submittedversion.
