# Supplementary material for: PtrA regulates prodigiosin synthesis and biological functions in Serratia marcescens FZSF02
Source: Front Microbiol. 2023 Sep 15;14:1240102. doi: 10.3389/fmicb.2023.1240102 (PMC10545897; doi:10.3389/fmicb.2023.1240102)
Supplement: Supplementary file 1 [file Image_1.pdf]

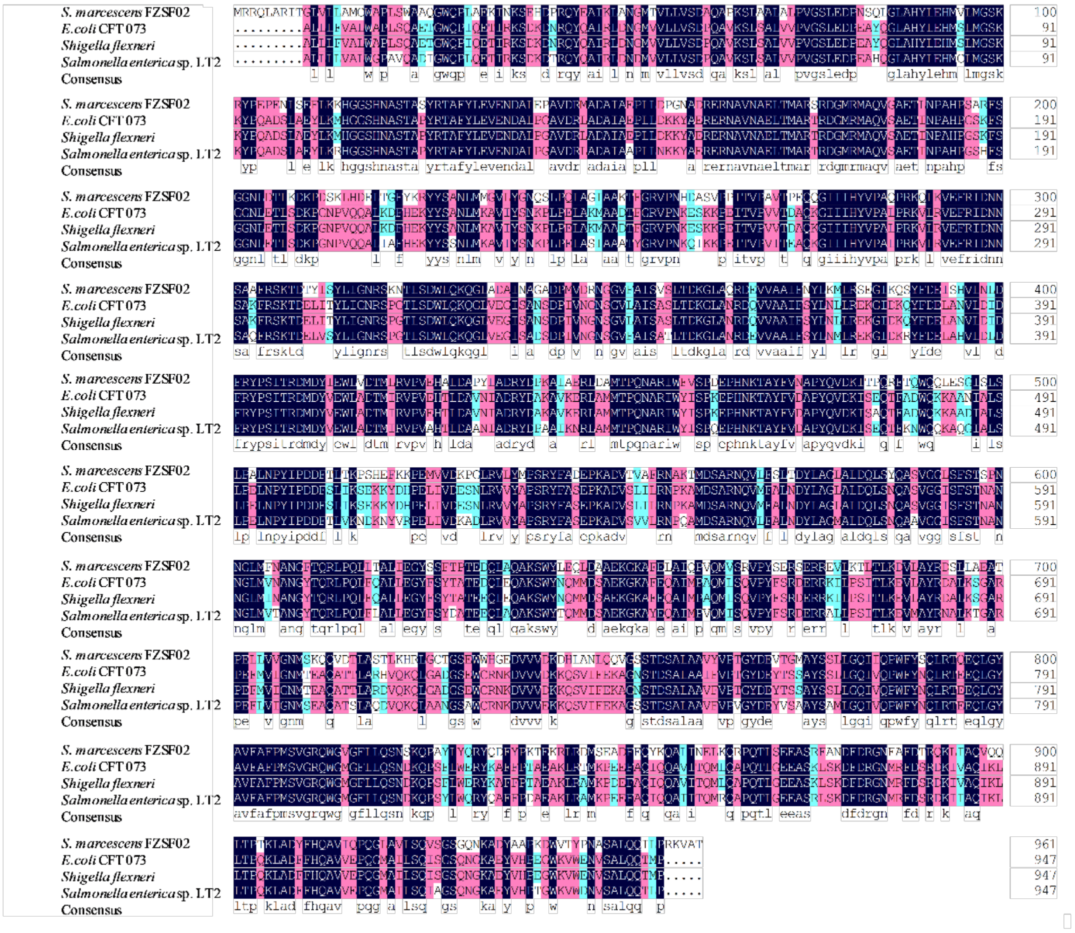

FIG S1 Sequence alignment of PtrA. Multiple sequence alignment of PtrA from *S. marcescens* FZSF02, *E. coli* CFT073, *Shigella flexneri*, *Salmonella enterica* sp. LT2.
